# Comparison of radiomics models and dual-energy material decomposition to decipher abdominal lymphoma in contrast-enhanced CT

**DOI:** 10.1007/s11548-023-02854-w

**Published:** 2023-03-06

**Authors:** Simon Bernatz, Vitali Koch, Daniel Pinto Dos Santos, Jörg Ackermann, Leon D. Grünewald, Inga Weitkamp, Ibrahim Yel, Simon S. Martin, Lukas Lenga, Jan-Erik Scholtz, Thomas J. Vogl, Scherwin Mahmoudi

**Affiliations:** 1 Department of Diagnostic and Interventional Radiology, University Hospital Frankfurt, Theodor-Stern-Kai 7, 60590 Frankfurt am Main, Germany; 2 Dr. Senckenberg Institute for Pathology, University Hospital Frankfurt, Goethe University Frankfurt am Main, 60590 Frankfurt am Main, Germany; 3 Department of Diagnostic and Interventional Radiology, University of Cologne, Faculty of Medicine, University Hospital Cologne, Kerpener Str. 62, 50937 Cologne, Germany; 4 Department of Molecular Bioinformatics, Institute of Computer Science, Johann Wolfgang Goethe-University, Robert-Mayer-Str. 11-15, 60325 Frankfurt am Main, Germany

**Keywords:** Radiomics, Artificial intelligence, Lymphoma, Oncology, DECT

## Abstract

**Purpose:**

The radiologists’ workload is increasing, and computational imaging techniques may have the potential to identify visually unequivocal lesions, so that the radiologist can focus on equivocal and critical cases. The purpose of this study was to assess radiomics versus dual-energy CT (DECT) material decomposition to objectively distinguish visually unequivocal abdominal lymphoma and benign lymph nodes.

**Methods:**

Retrospectively, 72 patients [*m*, 47; age, 63.5 (27–87) years] with nodal lymphoma (*n* = 27) or benign abdominal lymph nodes (*n* = 45) who had contrast-enhanced abdominal DECT between 06/2015 and 07/2019 were included. Three lymph nodes per patient were manually segmented to extract radiomics features and DECT material decomposition values. We used intra-class correlation analysis, Pearson correlation and LASSO to stratify a robust and non-redundant feature subset. Independent train and test data were applied on a pool of four machine learning models. Performance and permutation-based feature importance was assessed to increase the interpretability and allow for comparison of the models. Top performing models were compared by the DeLong test.

**Results:**

About 38% (19/50) and 36% (8/22) of the train and test set patients had abdominal lymphoma. Clearer entity clusters were seen in t-SNE plots using a combination of DECT and radiomics features compared to DECT features only. Top model performances of AUC = 0.763 (CI = 0.435–0.923) were achieved for the DECT cohort and AUC = 1.000 (CI = 1.000–1.000) for the radiomics feature cohort to stratify visually unequivocal lymphomatous lymph nodes. The performance of the radiomics model was significantly (*p* = 0.011, DeLong) superior to the DECT model.

**Conclusions:**

Radiomics may have the potential to objectively stratify visually unequivocal nodal lymphoma versus benign lymph nodes. Radiomics seems superior to spectral DECT material decomposition in this use case. Therefore, artificial intelligence methodologies may not be restricted to centers with DECT equipment.

**Supplementary Information:**

The online version contains supplementary material available at 10.1007/s11548-023-02854-w.

## Introduction

In patients with suspected lymphoma, suspicion of nodal involvement in cross-sectional imaging is primarily based on lymph node size and the number of nodes [1]. A high number of large lymph nodes increase the probability of malignancy [1]. To determine criteria for consistent assessment of pathological lymph nodes, several size thresholds were proposed [2]. Although determined thresholds exist, an accurate diagnosis of lymph node involvement in suspected or confirmed lymphoma still remains difficult in some cases. For example, when no prior CT is present or in cases with borderline lymph node size, further work-up may be required, potentially delaying diagnosis, therapy and prognosis [3, 4]. Positron emission tomography (PET) CT may confirm suspected nodal involvement, but high radiation exposure and costs require restrictive application [5]. Still, early identification of patients with lymphomatous disease is important and has prognostic relevance. As demand for medical imaging is steadily increasing, imaging biomarkers could serve as a supporting decision tool to counteract radiologists’ growing workload. Quantitative image analysis tools such as dual-energy CT (DECT) material decomposition analysis offer a non-invasive alternative to characterize tissue without the necessity of additional imaging and related radiation exposure. Some of these techniques have been extensively investigated over recent years [6, 7]. A commonly used post-processing technique is iodine-selective imaging. Iodine maps allow for further tissue characterization compared to subjective reporting and standard attenuation measurements in Hounsfield units (HU) [7, 8]. This material decomposition analysis technique is based on the differences in absorption characteristics for various elements at different energy levels [9, 10]. Thus, iodine quantification provides information about the local content of iodine contrast agent and can serve as a surrogate for tumor vascularity [11, 12]. The potential of DECT-based iodine quantification to differentiate benign and malignant lymph nodes has been assessed in recent studies [13–15].

Radiomics is a rapidly evolving research field that uses high-dimensional quantitative imaging features to describe tumor phenotypes objectively and quantitatively [16, 17]. Particularly in the field of oncology, the potential of radiomics features has been investigated as the features can provide additional, high-dimensional data [16]. Radiomics features capture tissue characteristics such as heterogeneity and shape and may be used for the prediction of tumor subtypes, treatment response and clinical outcome [16, 17]. The potential of DECT material decomposition and radiomics features for the characterization of benign and malignant lymph nodes including lymphoma has been assessed in recent studies [14, 15, 18, 19]. However, the combined application of both DECT and radiomics features for stratification of lymphoma has not been evaluated. It is unclear, which technique is superior and whether both techniques may support each other with increased predictive accuracy of a combined model.

In times of increasing demand for medical imaging, we hypothesized that quantitative imaging features may have the potential to serve as non-invasive imaging biomarkers to potentially stratify visually unequivocal lymphomatous lymph nodes. This approach may allow radiologists to focus on indistinct and borderline lymph nodes in the future [20]. In this study, we stratified and compared a set of robust and non-redundant radiomics features and DECT features to decipher abdominal lymphoma. We hypothesized that a combined application of radiomics and DECT features may improve the stratification of abdominal lymphoma in contrast-enhanced abdominal CT compared to DECT features only.

## Materials and methods

The local ethics committee approved this retrospective study (project number: 20–688 Goethe University Frankfurt am Main, Germany) and waived informed written consent.

### Study design

**Fig. 1:**
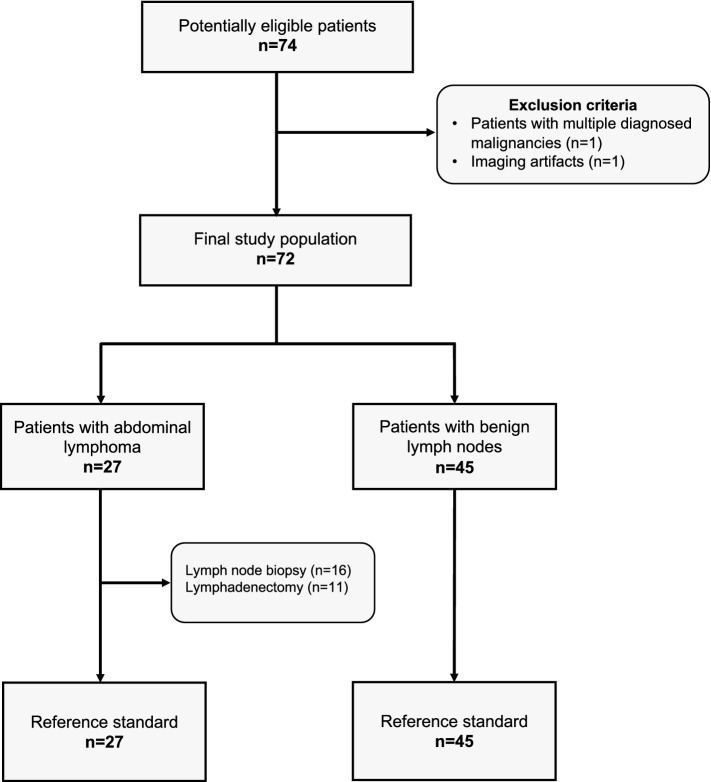
STARD flowchart of study inclusion

A total of 72 patients who had contrast-enhanced abdominal DECT imaging between 06/2015 and 07/2019 were included in the study. Inclusion criteria for the lymphoma cohort were as follows: (I) > 18 years of age, (II) visually unequivocal malignant lymph nodes > 1.5 cm [21], (III) confirmation of abdominal lymphoma by histopathology and (IV) abdominal DECT imaging with availability of 1.5 mm 100 kV and 150 kV series. Exclusion criteria were as follows: (I) multiple diagnosed malignancies and (II) imaging artifacts. The control cohort only comprised patients without diagnosis of abdominal malignancy. In cases of patients with multiple CT studies, the first DECT was used. All data were obtained in clinical routine. Figure 1 depicts the flowchart of patient inclusion following Standards for Reporting Diagnostic Accuracy Studies (STARD).

### CT acquisition protocol

All examinations were performed on a third-generation, dual-source, dual-energy CT (Somatom Force; Siemens Healthineers). The acquisition protocol operated the X-ray tubes at different kilovoltage and tube current settings (tube A: 100 kV, 190 mAs; tube B: 150 kV, 95 mAs). An additional tin filter (Selective Photon Shield II, Siemens Healthineers) was used in tube B to reduce radiation exposure. The dual-energy protocol (craniocaudal direction; rotation time, 0.5 s; pitch, 0.6; collimation, 2 × 192 × 0.6) included automatic attenuation-based tube current modulation (CARE Dose 4D; Siemens Healthineers). Contrast media injection was performed through a peripheral vein of the forearm at a flow of 2–3 ml/s. A non-ionic contrast agent (Imeron® 400 mg iodine/ml; Bracco, Milan, Italy) with a total of 1.2 ml/kg body weight (maximum of 120 ml) was administered. Image acquisition during venous phase of contrast enhancement started 70 s after contrast agent injection in inspiratory breath-hold. CT dose index (CTDI) and dose-length product (DLP) were recorded. Iterative reconstruction algorithm (ADMIRE®, Siemens Healthineers, Strength Level 3) was used for image reconstruction.

### Image preprocessing

DECT images were reconstructed in axial orientation (slice thickness, 1.5 mm; increment, 1.2 mm) with a dedicated dual-energy medium-soft convolution kernel [Qr40, advanced model-based iterative reconstruction (ADMIRE) level of 3]. DECT material decomposition image reconstruction was performed on a 3D multi-modality workstation (syngo.via, version VB10B, Siemens Healthineers). An iodine subtraction algorithm (Liver VNC, Siemens Healthineers) was used to calculate DECT data including iodine density (ID), normalized iodine uptake (ID%) and fat fraction. ID% was calculated: ID% = ID_lymph node_/ID_aorta_. Region-of-interest (ROI) ID_aorta_ was defined manually in the abdominal aorta at the level of the celiac trunk. For the radiomic analysis, the single-energy tube A image stack was exported in digital imaging and communications in medicine (DICOM) format and imported into the 3D Slicer software platform (http://slicer.org, version 4.9.0) [22, 23].

### Image segmentation

**Fig. 2:**
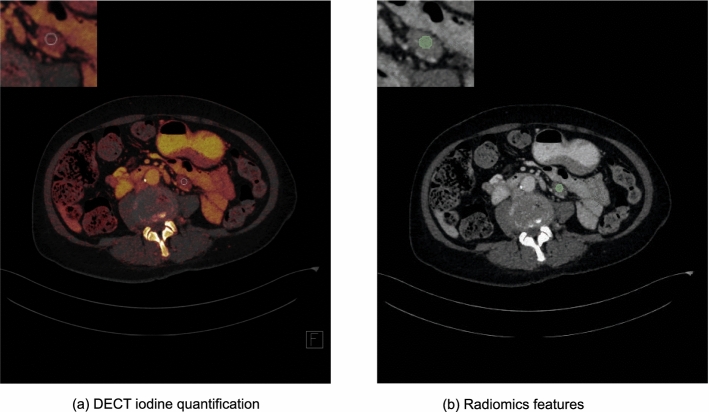
DECT iodine quantification and radiomics feature segmentation. A 66-year-old female patient with diffuse large B-cell lymphoma (DLBCL). Diagnosis was confirmed by lymphadenectomy and PET/CT. **a** Axial DECT-based iodine map image with region-of-interest (ROI) measurement of the respective lymph node. **b** Axial DECT image with standard volume-of-interest (VOI) measurement of the respective lymph node for radiomics feature extraction

One investigator (SM, radiologist in training, 2 years of experience) who had access to the clinical data (histopathology, PET-CT or follow-up imaging) chose and marked three lymph nodes per patient. ROI and spheric volume-of-interest (VOI) circumscription were performed manually by three blinded independent readers (I, SB, radiologist, in-training, 3.5 years of experience; II, VK, radiologist, in-training, 2 years of experience and III, IW, especially trained investigator, 1 year of experience). Each blinded investigator independently segmented one of the three previously marked lymph nodes. Priorly segmented lymph nodes were marked to exclude the segmentation of identical lymph nodes. In total, three segmentations, each of a different independent lymph node, were obtained per patient. ROI measurements for DECT analysis and VOI measurements for radiomics analysis were drawn as large as possible with a maximum diameter of 1.0 cm (Fig. 2), carefully avoiding surrounding structures, calcifications and visual artifacts. We chose a maximum diameter of 1.0 cm to exclude potential shape bias between enlarged lymphomatous and small benign lymph nodes. The segmentations were independently reviewed by a board-certified and blinded radiologist (SSM, 8 years of experience), and no disagreement was stated.

### Radiomics analysis

PyRadiomics v3.0.1 was used within the 3D Slicer software to extract radiomics features [23, 24] from the tube A single-energy images. With default settings, i.e., no resampling or filtering, bin width 25 and enforced symmetrical GLCM, we extracted all original standard features (*n* = 107, feature classes = 7) for each segmentation, as previously described [25]: shape, first-order statistics, gray level co-occurrence matrix (GLCM), gray level run length matrix (GLRLM), gray level size zone matrix (GLSZM), gray level dependence matrix (GLDM) and neighboring gray tone difference matrix (NGTDM) [25]. Shape features (*n* = 14) were excluded for the following analysis as we used spheric VOIs for feature extraction. The radiomics quality score was 12 (https://radiomics.world/rqs, Supplementary Material S1) [26].

### Interobserver robustness and analysis of feature redundancy

We calculated the intra-class correlation coefficient (ICC) for each feature and DECT material decomposition for the three independently segmented and measured lymph nodes per patient to assess the measurement’s stability [25]. ICC ranges from −1 (perfect anticorrelation) to 1 (perfect correlation), and we defined reproducibility with thresholds commonly used in radiomics studies: excellent (≥ 0.75), good (0.60–0.74), moderate (0.40–0.59) or poor ≤ 0.39 [25]. For further analysis, we discarded all radiomics features with ICC < 0.6 to include only radiomics features with at least good reproducibility (*n* = 31), and we excluded DECT features with ICC < 0.4 (iodine density) as no DECT feature had an ICC ≥ 0.6 (see Supplementary Data S2). We intercorrelated the robust features by Pearson method, and we excluded all highly correlated (Pearson > 0.95) redundant features (*n* = 13) (Supplementary Data S3).

### Radiographic biomarkers to predict lymphoma

We performed all analysis in Python 3.7.6, within Jupyter Notebook [27] and respective open-source packages. We aimed to predict our target variable (lymphoma) either using DECT material decomposition, combined DECT and radiomics features or radiomics features only. Therefore, we stratified our independent variables into three groups: I, DECT feature group; II, combined feature group and III, radiomics feature group. First, we performed explorative data analysis on our datasets using Euclidean distance matrices to explore the pairwise dataset relations and low-dimensional embedding with t-SNE plots to explore cohort distributions (scikit-learn [28]). Next, we used least absolute shrinkage and selection operator (LASSO) [28] to reduce the number of features and the risk of overfitting. LASSO did not select any DECT feature; therefore, the radiomics feature group and combined feature group yielded identical nonzero LASSO features, and we dismissed one redundant cohort (the combined feature group) for further analysis. The DECT feature group had only two features, and we did not perform LASSO for further feature reduction. We drew 70% random data samples as training set and used the remaining 30% to test our model. We locked the train and test split for all models. StandardScaler was used to scale the data to uniform variance. We trained and tested four individual and independent machine learning models [28]: (1) logistic regression classifier, (2) AdaBoost classifier, (3) gradient boosting classifier and (4) random forest classifier (see Supplementary Data S4 for detailed information on the feature selection and machine learning models). For each classifier, we calculated the mean importance score based on a 15 times shuffled permutation-based analysis of feature importance within the test set. We calculated the receiver operating characteristics (ROC) area under the curve (AUC), precision score and F-score for each model and depict the respective ROC curves.

### General statistics

We performed the statistical analyses in Python. For ICC analysis, we used the Pingouin package [29]. We used the implementation of the WORC.statistics package [30] for the DeLong’s test [31]. Graphical compositions were done in Affinity Designer 1.8.5.703 (Serif (Europe) Ltd.).

## Results

### Study population

The study population comprised 72 patients (*m*, 47; *f*, 25; median age, 63.5 (IQR, 57–64) years) with abdominal CT at a single DECT scanner. Twenty-seven patients (*m*, 15; *f*, 12; median age, 60 (IQR, 51–74) years) suffered from lymphoma; 38% (19/50) of the train set patients and 36% (8/22) of the test set patients. The diagnosis was made by histopathological analysis (lymph node biopsy, *n* = 16; lymphadenectomy, *n* = 11). The remaining patients (*n* = 45; *m*, 32; *f*, 13; median age, 67 (IQR, 59–74) years) did not have malignant abdominal lymph nodes and were part of the control cohort. Patient characteristics are depicted in Table 1.

**Table 1:** Patient characteristics

Parameters	All patients	Train set	Test set
Number of patients (n)	72	22	50
Male/female (n)	47/25	15/7	32/18
Mean age (years)	63.8 (27–87)	63.7 (35–86)	64.3 (27–87)
Patients with benign lymph nodes (*n*)	45	14	31
Patients with abdominal lymphoma (*n*)	27	8	19
Hodgkin lymphoma	4	2	2
Non-Hodgkin lymphoma (NHL)	23	6	17
B-cell lymphoma/T-cell lymphoma	19/4	4/2	15/2

If not depicted otherwise, the numbers without parenthesis depict absolute numbers. Data in round parenthesis are the min/max values

### Unsupervised cluster analysis

**Fig. 3:**
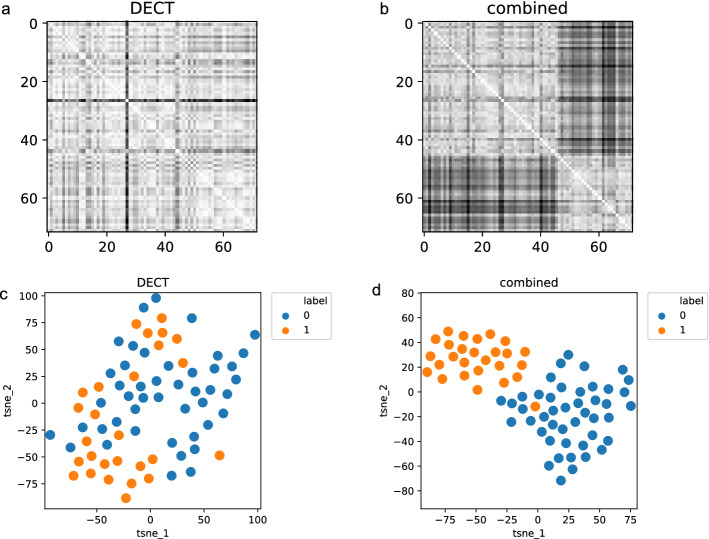
Unsupervised explorative data analysis. Euclidean distance matrices with samples sorted by label (lymphoma vs. benign) for the DECT (**a)** and combined feature cohort (**b**). White coloring depicts smaller Euclidean distances. Squarish patterns along the white diagonal show clusters with similar pairwise distance. In **c** and **d,** t-SNE plots of the DECT and combined cohort show the two-dimensional embedding of the joint probabilities (label: 1 (orange), lymphoma; 0 (blue), benign). *DECT* dual-energy computed tomography and *t-SNE* t-distributed stochastic neighbor embedding

The distance matrices depict the pairwise Euclidean distance for each sample. Using DECT features only, a clear stratification into two cohorts was not seen (Fig. 3a) whereas the combined feature group showed two unequivocally distinct clusters (Fig. 3b). The finding was corroborated in the respective t-SNE plots which depict lymphomatous lymph nodes in orange (label = 1) and benign lymph nodes in blue (label = 0). In the DECT feature group, no clear clustering was seen (Fig. 3c). Two clearly separated clusters were visualized in the combined feature group with only one outlier (Fig. 3d).

### Feature importance characteristics

We used LASSO to reduce the number of radiomics features from 18 robust and non-redundant features (ICC > 0.6 and Pearson correlation ≤ 0.95, see Supplementary Data S21 and S3) to our final radiomics feature set of five features (Table 2). The final features were part of the feature classes first order (*n* = 2), GLDM (*n* = 2) and GLSZM (*n* = 1). For each model, we calculated the importance of each feature for the final prediction. Within the DECT feature groups, fat fraction was superior to ID% in all models, and in two models (SGB and RF), ID% did not have any importance at all. In the radiomics feature group, one feature (large area high gray level emphasis) did show nonzero importance values in all models (Table 3).

**Table 2:** Least absolute shrinkage and selection operator (LASSO) feature selection

Feature	Class
Energy	First order
Maximum	First order
Dependence non-uniformity	GLDM
Large dependence emphasis	GLDM
Large area high gray level emphasis	GLSZM

The final feature set after LASSO feature reduction. *GLDM* gray level dependence matrix and *GLSZM* gray level size zone matrix

**Table 3:** Permutation-based feature importance analysis for each model

Radiomics	LR		AB		SGB		RF	
	Imp	Std	Imp	Std	Imp	Std	Imp	Std
Energy	0.030	0.027	0.000	0.000	0.021	0.023	0.303	0.036
Maximum	0.067	0.037	0.000	0.000	0.015	0.021	0.021	0.023
Dependence Non-uniformity	0.079	0.042	0.000	0.000	0.033	0.031	0.015	0.021
Large dependence Emphasis	0.118	0.040	0.000	0.000	0.000	0.000	0.000	0.000
Large area high Gray level emphasis	0.021	0.023	0.424	0.072	0.015	0.021	0.015	0.021
*DECT*								
Iodine_density_%	− 0.042	0.011	− 0.003	0.011	0.000	0.000	0.000	0.000
Fat_fraction	0.145	0.112	0.164	0.129	0.158	0.122	0.158	0.122

*LR* logistic regression, *AB* AdaBoost, *SGB* stochastic gradient boosting and *RF* random forest

### Model performance differences and best performing model comparison

**Fig. 4:**
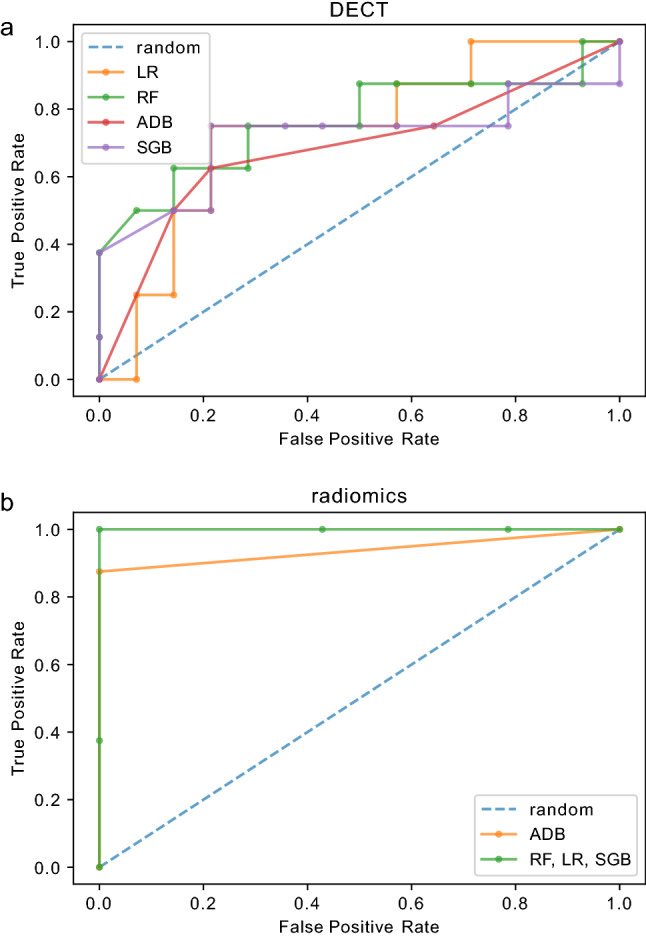
Performance visualization by receiver operating characteristics curves. Receiver operating characteristics (ROC) curves are depicted for the DECT (**a**) and radiomics (**b**) feature group. Each model is color coded. Models with identical performances are depicted in one color (see Table 4). *LR* logistic regression, *RF* random forest, *ADB* AdaBoost and *SGB* stochastic gradient boosting

**Fig. 5:**
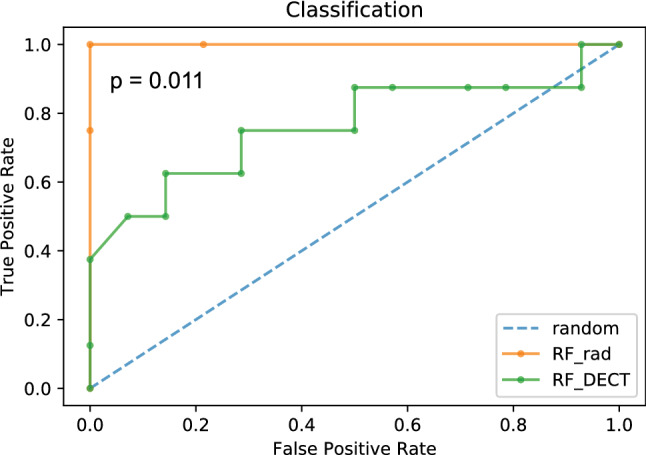
Comparison of the top performing models. Receiver operating characteristics (ROC) curves of the top performing model of the DECT (random forest, RF, green) and radiomics feature (random forest, RF, orange) group with DeLong test (*p* value) for statistical analysis

The DECT models achieved performances from AUC = 0.683 (*F*1 = 0.571, precision = 0.667) (ADB) up to AUC = 0.763 (*F*1 = 0.571, precision = 0.667, CI 0.435–0.923) (RF) (Fig. 4a). The radiomics models achieved performances from AUC = 0.938 (*F*1 = 0.933, precision = 1.000) (ADB) up to AUC = 1.000 (*F*1 = 1.000, precision = 1.000, CI 1.000–1.000) (LR, SGB and RF) (Fig. 4b). Detailed performance characteristics are depicted in Table 4. We compared the best performing models of the DECT (RF) and radiomics feature group (RF) model (Fig. 5). A significant superiority was seen for the radiomics feature group model (*p* = 0.011).

**Table 4:** Model performance metrics

	LR		AB		SGB		RF	
	Rad	DECT	Rad	DECT	Rad	DECT	Rad	DECT
AUC	1.000	0.732	0.938	0.683	1.000	0.714	1.000	0.763
F1	1.000	0.533	0.933	0.571	1.000	0.571	1.000	0.571
Prec	1.000	0.571	1.000	0.667	1.000	0.667	1.000	0.667

*AUC* area under the curve, *LR* logistic regression, *AB* AdaBoost, *SGB* stochastic gradient boosting, *rad* radiomics and *RF* random forest

## Discussion

In this retrospective study, we evaluated the potential of radiomics features to stratify visually unequivocal abdominal lymphoma in comparison to DECT-based material decomposition analysis. The findings of our study suggest superior performance of radiomics-based machine learning models compared to DECT-based material decomposition analysis techniques for the stratification of abdominal lymphoma in contrast-enhanced abdominal CT. Thus, quantitative image biomarkers may not be restricted to centers with DECT equipment as artificial intelligence methodologies including radiomics are applicable in standard single-energy CT. Our findings indicate that the automatic stratification of unequivocal lymphomatous lymph nodes based on radiomics features may be feasible, and it could serve as a prioritization support tool to help radiologists focus on critical cases.

Over recent years, DECT post-processing techniques and radiomics have become rapidly evolving research fields in cancer research, leading to improved non-invasive lesion characterization [16, 32, 33]. The ability of DECT to provide information about lymph node characterization beyond subjective evaluation and simple attenuation measurements has been validated in prior studies [13–15]. In a study from 2018, the authors investigated the potential of DECT iodine quantification to differentiate lymphoma, lymph node metastases and benign lymph nodes [13]. Whereas significant differences were found between lymphoma and lymph node metastases, a discrimination between lymphoma and benign lymph nodes could not be demonstrated using DECT iodine quantification. Therefore, prediction of lymphoma in CT using DECT-based imaging biomarkers has not yet been approved. In contrast to basic material decomposition analysis techniques, radiomics can provide additional, higher dimensional data by extracting a variety of mineable image features [16]. Several studies have investigated the impact of radiomics for tissue and tumor characterization. In the field of oncologic imaging, the potential of radiomics for the stratification of lymph node metastases has been shown for several tumor entities, such as breast cancer, colorectal carcinoma, gastric cancer and lung cancer [34–37]. All of these studies have demonstrated the ability of radiomics features to stratify lymph node metastases in a preoperative setting. Similar findings have been presented for the identification of lymphoma using radiomics features [38]. In multiple studies, the predictive performance of CT-derived radiomics features has been successfully validated for several lymphoma subtypes, including Hodgkin lymphoma, diffuse large B-cell lymphoma, mantle-cell lymphoma and follicular lymphoma [38–41]. The novelty of our study relates to the direct comparison of the accuracy of DECT material decomposition versus radiomics features to differentiate benign and lymphomatous lymph nodes non-invasively. Further, we aimed to analyze the feasibility to objectively stratify visually unequivocal cases to path the way for a potential flagging tool, so that radiologists can focus their workforce on equivocal or critical cases. We could demonstrate superior predictive performance of machine learning models trained with radiomics features compared to DECT material decomposition values. Our findings demonstrate higher robustness of radiomics features compared to DECT material decomposition which is an important factor for sustainable research. One major advantage of radiomics compared to DECT-based algorithms is that radiomics can be applied on standard-of-care CT scans without the necessity for DECT equipment. DECT is not as widespread in health centers as single-energy CT. Therefore, DECT-based material decomposition analysis techniques are restricted to a minor group of well-equipped health-care centers. In sharp contrast to DECT-based material decomposition, CT-based radiomics features offer a superior quantitative data characterization tool that is accessible to a very wide hospital spectrum. Even in health centers where DECT equipment is available, retrospective application of DECT post-processing is limited to scans in which DECT raw data (1.5 mm low and high kV series) are available as DECT raw data are mandatory for post-processing. Due to storage capacity reasons, these DECT raw data are not always available for material decomposition analysis reconstruction. This limitation restricts the use of DECT material decomposition analysis to selected DECT scans. As a consequence, in the screening process of the current study, a major part of potentially eligible cases had to be excluded due to incomplete DECT raw data. Our study has further limitations, which have to be taken into account. We analyzed retrospective data. Therefore, we cannot rule out selection bias. Also, our study included 72 patients, and a larger cohort might have been favorable. This might reduce generalizability of the result. Last, we restricted the patient inclusion to one dual-energy CT scanner to exclude inter-scanner variability and to include only reconstructions with a slice thickness of 1.5 mm and increment of 1.2 mm; nevertheless, intra-scanner variability may have occurred.

In conclusion, our findings indicate that radiomics features are superior to DECT material decomposition for the objective identification of visually unequivocal abdominal lymphoma in contrast-enhanced CT scans. In patients with suspected or diagnosed abdominal lymphadenopathy, radiomics may assist in clinical diagnosis as a support tool for the identification of lymphomatous lymph nodes. In times of steadily increasing radiological workload, radiomics and DECT-based biomarkers may be used to identify visually unequivocal lymph nodes, promoting that radiologists can focus on equivocal and critical cases. In medical institutions without DECT equipment, radiomics-based machine learning models may offer an alternative for the non-invasive prediction of abdominal lymphoma with superior diagnostic accuracy compared to DECT material decomposition analysis.

## Supplementary Information

Below is the link to the electronic supplementary material.

Supplementary file1 (DOCX 606 kb)

## Data Availability

Data are available upon reasonable request from the corresponding author.

## Abbreviations

AUC: Area under the curve
CT: Computed tomography
CTDI: CT dose index
DECT: Dual-energy computed tomography
DICOM: Digital imaging and communications in medicine
DLP: Dose-length product
GLCM: Gray level co-occurrence matrix
GLDM: Gray level dependence matrix
GLRLM: Gray level run length matrix
GLSZM: Gray level size zone matrix
HU: Hounsfield units
ICC: Intra-class correlation coefficient
ID: Iodine density
ID%: Normalized iodine uptake
IQR: Interquartile range
LASSO: Least absolute shrinkage and selection operator
NGTDM: Neighboring gray tone difference matrix
PET: Positron emission tomography
ROC: Receiver operating characteristics
ROI: Region of interest
STARD: Standards for Reporting Diagnostic Accuracy Studies
