# Supplementary material for: Comparison of radiomics models and dual-energy material decomposition to decipher abdominal lymphoma in contrast-enhanced CT
Source: Int J Comput Assist Radiol Surg. 2023 Mar 6;18(10):1829–39. doi: 10.1007/s11548-023-02854-w (PMC10497439; doi:10.1007/s11548-023-02854-w)
Supplement: Supplementary file 1 — Supplementary file1 (DOCX 606 kb) [file 11548_2023_2854_MOESM1_ESM.docx]

**Comparison of radiomics models and dual-energy material decomposition to decipher abdominal lymphoma in contrast-enhanced CT**

***Supplementary Material***

Simon Bernatz, MD^1,2^; Vitali Koch, MD^1^; Daniel Pinto Dos Santos, MD^1,3^; Jörg Ackermann, PhD^4^, Leon D. Grünewald, MD^1^; Inga Weitkamp, Ms^1^; Ibrahim Yel, MD^1^; Simon S. Martin, MD^1^; Lukas Lenga, MD^1^; Jan-Erik Scholtz, MD^1^; Thomas J. Vogl, MD^1^; Scherwin Mahmoudi, MD^1^

^1^Department of Diagnostic and Interventional Radiology, University Hospital Frankfurt, Goethe University Frankfurt am Main, Theodor-Stern-Kai 7, 60590 Frankfurt am Main, Germany;

^2^Dr. Senckenberg Institute for Pathology, University Hospital Frankfurt, Goethe University Frankfurt am Main, 60590, Frankfurt am Main, Germany;

^3^Department of Diagnostic and Interventional Radiology, University of Cologne, Faculty of Medicine and University Hospital Cologne, Kerpener Str. 62, 50937, Cologne, Germany;

^4^Department of Molecular Bioinformatics, Institute of Computer Science, Johann Wolfgang Goethe-University, Robert-Mayer-Str. 11-15, 60325 Frankfurt am Main, Germany.

[**S1. Radiomics quality score** 3](#_Toc104838341)

[**S2. Intra-class correlation analysis** 6](#_Toc104838342)

[**S3. Pearson correlation analysis** 10](#_Toc104838343)

[**S4. Machine learning** 11](#_Toc104838344)

# **S1. Radiomics quality score**

# **S2. Intra-class correlation analysis**

*Supplementary Figure 1*: Box-whisker plots of ICC3 values grouped by feature class.

*Supplementary Figure 2*: Swarmplot of the ICC3 value of each individual radiomic feature.

*Supplementary Figure 3*: Swarmplot of ICC3 values of DECT material decomposition

| **class** | **features** | **ICC3** |
| --- | --- | --- |
| glrlm | RunVariance | 0.882 |
| glrlm | LongRunEmphasis | 0.876 |
| gldm | LargeDependenceEmphasis | 0.861 |
| glrlm | RunPercentage | 0.855 |
| glrlm | GrayLevelNonUniformity.1 | 0.831 |
| gldm | GrayLevelNonUniformity | 0.830 |
| glrlm | ShortRunEmphasis | 0.829 |
| gldm | DependenceEntropy | 0.828 |
| glrlm | RunLengthNonUniformityNormalized | 0.828 |
| glrlm | RunLengthNonUniformity | 0.819 |
| gldm | DependenceNonUniformity | 0.808 |
| glszm | LargeAreaEmphasis | 0.797 |
| glszm | ZoneVariance | 0.795 |
| glszm | GrayLevelNonUniformity.2 | 0.792 |
| firstorder | Energy | 0.788 |
| firstorder | TotalEnergy | 0.783 |
| gldm | DependenceVariance | 0.730 |
| glszm | ZonePercentage | 0.728 |
| glrlm | RunEntropy | 0.715 |
| glcm | Idn | 0.712 |
| glszm | ZoneEntropy | 0.679 |
| glszm | LargeAreaLowGrayLevelEmphasis | 0.678 |
| ngtdm | Coarseness | 0.675 |
| glszm | LargeAreaHighGrayLevelEmphasis | 0.674 |
| glszm | SizeZoneNonUniformity | 0.672 |
| glcm | Idmn | 0.665 |
| gldm | SmallDependenceEmphasis | 0.649 |
| glcm | Imc1 | 0.628 |
| glcm | SumEntropy | 0.623 |
| firstorder | Maximum | 0.620 |
| glcm | Imc2 | 0.617 |
| **glcm** | **JointEntropy** | **0.587** |
| **gldm** | **SmallDependenceLowGrayLevelEmphasis** | **0.571** |
| **gldm** | **DependenceNonUniformityNormalized** | **0.567** |
| **glcm** | **MCC** | **0.551** |
| **ngtdm** | **Strength** | **0.545** |
| **glcm** | **Correlation** | **0.542** |
| **glcm** | **JointEnergy** | **0.539** |
| **firstorder** | **90Percentile** | **0.511** |
| **gldm** | **LargeDependenceHighGrayLevelEmphasis** | **0.510** |
| **glcm** | **DifferenceEntropy** | **0.502** |
| **firstorder** | **Mean** | **0.500** |
| **ngtdm** | **Busyness** | **0.492** |
| **firstorder** | **Median** | **0.492** |
| **firstorder** | **10Percentile** | **0.447** |
| **glrlm** | **ShortRunLowGrayLevelEmphasis** | **0.444** |
| **firstorder** | **RootMeanSquared** | **0.434** |
| **firstorder** | **Range** | **0.434** |
| **glcm** | **MaximumProbability** | **0.400** |
| **glszm** | **SmallAreaHighGrayLevelEmphasis** | **0.394** |
| **glszm** | **GrayLevelVariance.2** | **0.377** |
| **ngtdm** | **Contrast.1** | **0.369** |
| **glrlm** | **LowGrayLevelRunEmphasis** | **0.366** |
| **glcm** | **Id** | **0.365** |
| **glrlm** | **LongRunHighGrayLevelEmphasis** | **0.363** |
| **glcm** | **Idm** | **0.354** |
| **gldm** | **LowGrayLevelEmphasis** | **0.352** |
| **glszm** | **HighGrayLevelZoneEmphasis** | **0.333** |
| **glszm** | **LowGrayLevelZoneEmphasis** | **0.319** |
| **glcm** | **DifferenceAverage** | **0.308** |
| **ngtdm** | **Complexity** | **0.274** |
| **glszm** | **SmallAreaEmphasis** | **0.274** |
| **firstorder** | **Minimum** | **0.261** |
| **gldm** | **SmallDependenceHighGrayLevelEmphasis** | **0.249** |
| **glcm** | **Contrast** | **0.242** |
| **firstorder** | **Entropy** | **0.227** |
| **glrlm** | **GrayLevelNonUniformityNormalized** | **0.224** |
| **glszm** | **SizeZoneNonUniformityNormalized** | **0.219** |
| **glcm** | **SumAverage** | **0.213** |
| **glcm** | **JointAverage** | **0.213** |
| **firstorder** | **Kurtosis** | **0.210** |
| **firstorder** | **Uniformity** | **0.200** |
| **glszm** | **GrayLevelNonUniformityNormalized.1** | **0.200** |
| **glcm** | **DifferenceVariance** | **0.187** |
| **gldm** | **LargeDependenceLowGrayLevelEmphasis** | **0.164** |
| **glrlm** | **HighGrayLevelRunEmphasis** | **0.150** |
| **gldm** | **HighGrayLevelEmphasis** | **0.131** |
| **glcm** | **Autocorrelation** | **0.126** |
| **glcm** | **SumSquares** | **0.121** |
| **gldm** | **GrayLevelVariance** | **0.114** |
| **glrlm** | **ShortRunHighGrayLevelEmphasis** | **0.113** |
| **glrlm** | **LongRunLowGrayLevelEmphasis** | **0.113** |
| **glcm** | **ClusterTendency** | **0.110** |
| **firstorder** | **Skewness** | **0.107** |
| **glrlm** | **GrayLevelVariance.1** | **0.101** |
| **glszm** | **SmallAreaLowGrayLevelEmphasis** | **0.095** |
| **firstorder** | **MeanAbsoluteDeviation** | **0.088** |
| **firstorder** | **RobustMeanAbsoluteDeviation** | **0.085** |
| **firstorder** | **Variance** | **0.080** |
| **firstorder** | **InterquartileRange** | **0.018** |
| **glcm** | **ClusterProminence** | **0.006** |
| **glcm** | **ClusterShade** | **-0.052** |
| **glcm** | **InverseVariance** | **-0.053** |

*Supplementary Table 1*: Sorted ICC3 values of each radiomic feature. Marked in bold are features with ICC3 < 0.6 which were excluded for further analysis.

| **features** | **ICC3** |
| --- | --- |
| **Iodine Density (mg/ml)** | **0.218** |
| I.D. % | 0.403 |
| Fat Fraction | 0.477 |

*Supplementary Table 2*: ICC3 values for DECT material decomposition features. Feature marked in bold was excluded with ICC < 0.4 (poor).

# **S3. Pearson correlation analysis**

*Supplementary Figure 4*: Pearson correlation heatmap of robust (ICC3 ≥ 0.6) radiomic features

Highly correlated features (Pearson correlation ≥ 0.95): *TotalEnergy; Idn;; GrayLevelNonUniformity[gldm]; GrayLevelNonUniformity.1[glrlm]; LongRunEmphasis; RunLengthNonUniformity; RunLengthNonUniformityNormalized; RunPercentage; RunVariance; ShortRunEmphasis; GrayLevelNonUniformity.2[glszm]; LargeAreaEmphasis; ZoneVariance* were dropped to reduce feature redundancy.

# **S4. Machine learning**

*Feature reduction*: We used Least Absolute Shrinkage and Selection Operator (LASSO) as implemented in scikit-learn 1.0.2 (1) to reduce the high dimensional feature space. We applied the class sklearn.linear_model.Lasso with default values (alpha=1.0, fit_intercept=True, normalize='deprecated', precompute=False, copy_X=True, max_iter=1000, tol=0.0001, warm_start=False, positive=False, random_state=None, selection='cyclic'). We selected the non-zero features as inputs for our machine learning model pool.

*Dataset preparation*: The scaled and selected features were split into training (70%) and testing (30%) datasets. We took the same data-split to train and test all our machine learning models to exclude the impact of variant splits on the machine learning performance and feature importance.

*Machine learning model pool*: We applied four variant machine learning models composed of a logistic regression classifier, random forest classifier, AdaBoost Classifier and Gradient Boosting Classifier as implemented in scikit-learn 1.0.2 (1). We defined a constant seed for the random_sate for all models to exclude seed-induced inter-model differences.

*Logistic Regression*: Class sklearn.linear_model.LogisticRegression with default values: penalty='l2', dual=False, tol=0.0001, C=1.0, fit_intercept=True, intercept_scaling=1, class_weight=None, max_iter=100, multi_class='auto', verbose=0, warm_start=False, n_jobs=None, l1_ratio=None and specification of solver = “liblinear” as we face a small dataset.

*AdaBoost Classifier*: Class sklearn.ensemble.AdaBoostClassifier with default values: learning_rate=1.0, algorithm='SAMME.R' with specified n_estimators=100 and DecisionTreeClassifier with a max_depth = 5 as base_estimator

*Gradient Boosting Classifier*: Class sklearn.ensemble.GradientBoostingClassifier with default values loss='deviance', learning_rate=0.1, n_estimators=100, criterion='friedman_mse', min_samples_split=2, min_samples_leaf=1, min_weight_fraction_leaf=0.0, min_impurity_decrease=0.0, init=None, random_state=None, verbose=0, max_leaf_nodes=None, warm_start=False, validation_fraction=0.1, n_iter_no_change=None, tol=0.0001, ccp_alpha=0.0 and specified max_depth=1, subsample=0.8, max_features=0.2.

*Random Forest Classifier*: Class sklearn.ensemble.RandomForestClassifier with default values n_estimators=100, *, criterion='gini', min_samples_split=2, min_samples_leaf=1, min_weight_fraction_leaf=0.0, max_features='auto', max_leaf_nodes=None, min_impurity_decrease=0.0, bootstrap=True, oob_score=False, n_jobs=None, random_state=None, verbose=0, warm_start=False, class_weight=None, ccp_alpha=0.0, max_samples=None and specified max_depth of 5.

**S5. References**

1. Pedregosa F, Varoquaux G, Gramfort A, et al. Scikit-learn : Machine Learning in Python. J Mach Learn Res. 2011;12:2825–2830.
